# Supplementary material for: Learning curve and surgical outcome of robotic assisted colorectal surgery with ERAS program
Source: Sci Rep. 2022 Nov 29;12:20566. doi: 10.1038/s41598-022-24665-w (PMC9709162; doi:10.1038/s41598-022-24665-w)
Supplement: Supplementary file 2 — Supplementary Information 2. [file 41598_2022_24665_MOESM2_ESM.zip › RAL Raw data-Ñ[▒K/101/OP N_ñw╝╨░O▒Kñσ.pdf]

W52 / W52

臺中榮民總醫院
手術室護理記錄

[redacted]

基本資料

- 室別： 開刀房-17室，手術科別： CRS，手術類別： 常規，日期： 2021/07/28
- 進入時間： 0755 ； 麻醉開始時間： 0817 ； Time Out時間： 1010 ； 劃刀時間： 1010 ； 手術結束時間： 1530 送出時間： 1550 ；
- 麻醉方式： GE

  診斷：rectum ca s/p ccrt

  手術名稱：Robot Assisted Low Anterior Resection

  手術局部用藥：NIL

  術前預防性抗生素：
  - 抗生素名稱一： Veterin 1GM IVD 由麻醉護理人員協助執行
  - 時間： 0925
  - 上次簽章: 蘇敬涵

  ，追加預防性抗生素：
  - 抗生素名稱一： Veterin 1GM IVD由麻醉護理師給予
  - 時間： 1325
  - 上次簽章: 王明盈
- 手術醫師： 林俊余，林陳彥，陳映菁
- 麻醉醫師： VS 陳永銘
-
  - 刷手護士A ： 歐真勤/廖娸?，時間： 0755，刷手護士B ： 王明盈，時間： 0755，接替刷手一： 歐真勤，時間： 1200，接替刷手二： 歐真勤/廖娸?，時間： 1230
  - 流動護士A ： 蘇敬涵，時間： 0755，接替流動一： 王明盈，時間： 1200，接替流動二： 李郁萱，時間： 1500
- 更多接替及流動護士： 接替刷手三： 邱欐婇/廖娸?，時間： 1300，接替刷手四： 邱欐婇/蘇敬涵，時間： 1500，接替刷手五： 蘇敬涵，時間： 1530

皮膚狀況

A-V Shunt： 無

術前： 不完整

部位(術前)：

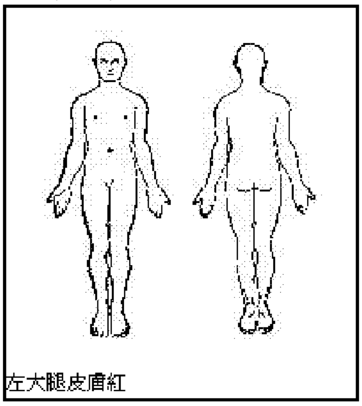

記錄者： 上次簽章: 蘇敬涵

術後： 不完整

部位(術後)：

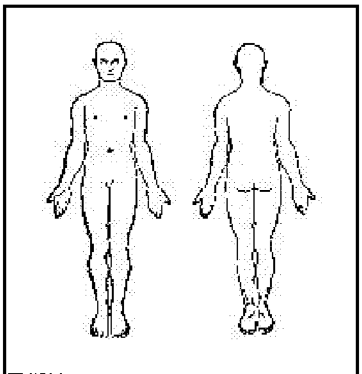

記錄者： 上次簽章: 李郁萱

手術準備

手術臥式 單一手術臥式

-
  - 手術臥式： 截石臥式
  - 使用附件： 溫毯 38 ℃，腳架，其它： CRS 輔助用物
  - 皮膚準備： 10%Aq Betadine，Hibiscrub，2% Chlorhexidine
  - 電燒： 使用電燒: 電極片位置： 其它： 迴路電極墊，記錄者： 上次簽章: 蘇敬涵

止血帶T： 無

植入物

無

標本送檢

- 細菌培養 無
- 細胞學 無
- 冰凍切片 無
- 病理標本
  組織名稱一：Rectum
  ,
  組織名稱二：IMA root
  ,
  組織名稱三：Distal cut end
- 截肢 無

標本交付 標本： 3 袋，標本： 0 盒，標本： 0 瓶，細胞學： 0 管，Culture Tube： 0 管，被交付醫師： CR 林陳彥，

- 記錄者： 上次簽章: 李郁萱
- 時間： 1540

傷口術後敷料

傷口敷料： 傷口敷料： 無

- 請於圖上標示部位：

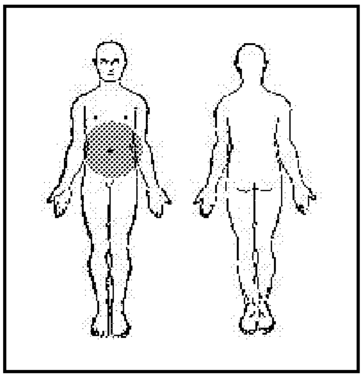

- 傷口敷料： Mepore、白紗

導管

導管	天數	導管資訊	插入日期	插入時間	更換日期	拔除日期	拔除時間	拔除原因	預計拔除/更換	預計拔除/更換日期
Double Lumen CVC(1)	1	號碼:8 Fr.; 深度:15cm; 部位:jugular (右); 象限:RU;	2021/07/28	0858						
Foley(1)	1	號碼:16 Fr.; 部位:膀胱;	2021/07/28	0858						
Penrose Drain(1)	1	號碼:12 mm.; 部位:骨盆腔;	2021/07/28	1500						
A-line (1)	1	號碼:20 G; 部位:左手;	2021/07/28	0858						
Chemo Port(1)	89	部位:鎖骨下; 象限:LU;	2021/05/01	1000						

用物計數

- 手術中出血量： MINIMAL c.c.
- 手術中尿量： 300 c.c.
-
    - 其它： 無

用物計數： 多切口同時縫合

- 1.器械： 正確，2.縫針： 正確，3.刀片： 正確，4.紗布： 正確，5.腹部墊： 未使用，6.紗球： 未使用，7.PEANUTS ： 未使用，8.紗條： 未使用，9.絲帶： 未使用，10.針頭： 未使用，11.Vessel loop： 未使用，12.Boots： 未使用，13.Jelco： 未使用，14.棉條： 未使用，
    - 第一次：
        - 刷手護士 ： 邱欐媃/廖娸?
        - 流動護士： 上次簽章: 王明盈
        - 時間： 1425

  ,
    - 第二次：
        - 刷手護士 ： 邱欐媃/廖娸?
        - 流動護士： 上次簽章: 王明盈
        - 時間： 1450

  ,
    - 第三次：
        - 刷手護士 ： 邱欐媃/廖娸?
        - 流動護士： 上次簽章: 王明盈
        - 時間： 1505

備註

備註： R17 Davinci 30度 Scope NO.1
R17 Davinci 0度 Scope NO.2

- 記錄者： 上次簽章: 李郁萱
- 時間： 1550

填寫護理人員 李郁萱

正式摘要

W52 / W52

doctorRank:B
